# Knowledge, Attitude, and Practice Toward Cardiovascular Diseases in the Lebanese Population

**DOI:** 10.5334/gh.1138

**Published:** 2022-07-29

**Authors:** Marc Machaalani, Battoul Fakhry, Maisoon Zwaideh, Karl Mendelek, Nadine Mahmoud, Talal Hammoud, Mirna N. Chahine

**Affiliations:** 1 Faculty of Medical Sciences, Lebanese University, Hadath, Lebanon; 2 Beirut Cardiac Institute, Al-Rassoul Al-Aazam Hospital, Beirut, Lebanon; 3 Basic Sciences Department, Faculty of Medical Sciences, Lebanese University, Hadath, Lebanon; 4 Foundation-Medical Research Institutes (F-MRI®), Beirut, Lebanon/Geneva, Switzerland

**Keywords:** CVD (cardiovascular diseases), KAP (knowledge; Attitude; Practice), Awareness

## Abstract

**Background & Objective:**

Cardiovascular diseases (CVD) are the leading cause of death globally. Assessing CVD knowledge, attitude, and practice (KAP) is necessary to spread awareness about CVD in Lebanon, their corresponding risk factors, and behaviors in which individuals can avoid or minimize the possibility of developing a CVD.

**Subjects & Methods:**

This was a case-control analytical study that targeted 921 CVD and non-CVD subjects. A questionnaire form was used to collect data related to patients’ demographics, socioeconomic status, habits, medical and family history, KAP towards CVD, and source of information. Data was analyzed using SPSS v.25.

**Results:**

Data from 921 participants were distributed over the CVD group (52.6% males aged 58.3 ± 13.7 years [n = 460]) and the non-CVD group (47.7% males aged 36.3 ± 15.4 years [n = 461]). CVD patients were significantly older than non-CVD subjects (p < 0.001). All three KAP scores of both groups were of poor to fair levels. Both CVD knowledge and attitude mean scores in CVD patients (26.6 ± 5.2 over 40 [66.50%] and 63.3 ± 10.2 over 85 [74.47%], respectively) were significantly higher than the ones of non-CVD subjects (23.5 ± 7.9 over 40 [58.75%] and 61.4 ± 12.4 over 85 [72.74%], respectively, p < 0.001). However, the CVD mean practice score was significantly lower in CVD patients (6.0 ± 1.7 over 9 [66.67%]) than the one of non-CVD subjects (6.3 ± 2.2 over 9 [70.00%] p < 0.001). Mostly, educational level (p < 0.001), governorate (p < 0.01), and smoking (p < 0.001) were predictors of KAP CVD in both groups.

**Conclusion:**

With an overall limited knowledge, attitude, and practice toward CVDs, the Lebanese population (with CVD or non-CVD) needs targeted national campaigns about CVD according to the identified predictors of CVD KAP to prevent and to alleviate the complications due to CVDs.

## Introduction

Cardiovascular diseases (CVDs) comprise various congenital or acquired diseases that affect the heart or blood vessels [1]. According to the World Health Organization (WHO), CVDs are the major cause of mortality worldwide, resulting in 17.9 million global deaths annually [2,3]. They are also considered to be among the most costly medical conditions [4], and prevention strategies are needed to limit the high prevalence of CVDs and mitigate their costs [5].

The American Heart Association measures cardiovascular health by seven factors: physical activity, smoking status, blood sugar level, body weight, cholesterol level, blood pressure, and diet [6]. Non-modifiable risk factors include age, sex, family history, and race, whereas modifiable risk factors are high blood pressure, dyslipidemia, smoking, diabetes, obesity, sedentary lifestyle, unhealthy diet, and stress [7,8,9]. Such risk factors may predispose to a wide range of complications such as coronary artery disease, cardiac dysrhythmias, cerebrovascular disease, cardiomyopathies, and peripheral vascular disease [10], hence the importance to control modifiable risk factors. In Lebanon, for instance, there is a great need to raise awareness about CVD risk factors as demonstrated by Fahs et al. in 2017 on 1,000 Lebanese participants who showed a higher prevalence of cardiovascular risk factors [11].

Management of CVDs involves on one hand non-pharmacological interventions such as: nutritional education [12], physical activity [13], lowering the Body Mass Index (BMI) and maintaining a healthy weight [14], reduction of salt intake [15], and smoking cessation [16,17,18]. On the other hand, pharmacological treatments include angiotensin-converting enzyme inhibitors (ACEIs), angiotensin receptor blockers (ARBs), aldosterone antagonists, loop diuretics, beta-blockers, anti-hypertensive drugs, anti-diabetic medications, lipid lowering agents, oral anticoagulants, and anti-platelet agents [19,20,21,22]. Despite the progress in CVD management, prevalence of CVD in the world [23], and Lebanon (36%) [11] remain high, and 47% of deaths in Lebanon in 2016 were due to CVD.

One way for controlling risk factors and decreasing prevalence rate of CVD is through primary prevention, early diagnosis, and good CVD practices. Therefore, to raise CVD awareness, February is recognized as the ‘American Heart Month’ through various activities [24]. Astoundingly, in the last 50 years, the death rate due to CVD decreased by 70% [25]. However, despite such educational campaigns, poor awareness about CVD is still prevalent, as revealed by various studies that aimed to assess the level of general CVD knowledge, attitude, practice (KAP), including one on obese Latina women [26], and others in Malaysia [27,28] and Iran [29]. For instance, findings by Aminde et al. in Cameroon revealed that women had poor knowledge about CVD, and that high education level, high monthly income, having a family history of CVD, and being a former smoker were associated to moderate-to-good knowledge [30]. In Lebanon, a cross-sectional study conducted by Ghaddar et al. showed that individuals had a moderate-to-high degree of knowledge about cardiovascular risk factors, but a low-to-moderate level of adherence toward CVD management, such as physical exercise, weight loss, and smoking cessation [31].

Since KAP studies about CVDs are getting scant attention, specifically in Lebanon and in Arab countries, and that only one study was conducted to assess KAPs of CVD among non-cardiac Lebanese individuals [31], we recommend further research in this area. Consequently, this cross-sectional study aimed to assess KAP levels toward CVD among the Lebanese population with CVD subjects and non-CVD subjects (control), while identifying the factors affecting KAP CVD scores and the socio-demographic characteristics predicting these scores, in order to spread awareness and prevent CVDs.

## Methods

### 1. Ethical information

Before we had started our study, we received an IRB waiver from the ethical committee of the Hayat Hospital (Reference Number: ETC-11-2021). This study was conducted in accordance with Good Clinical Practice ICH Section three, and the principles laid down by the 18th World Medical Assembly (Helsinki, 1964) and all applicable amendments. This study was confidential as each filled survey was associated with a number, thereby ensuring that all identities remain anonymous. Participants were asked to sign electronically an informed consent if they agree to participate voluntarily in our study. The study participants received detailed explanation of the background, objectives, risks, and advantages of the study and they were clearly informed of their right to withdraw at any time and that the information they provided was treated confidentially.

### 2. Study design

This study was a cross-sectional survey conducted from July 4^th^ till August 4^th^ 2021 to assess KAP towards CVD using an electronic survey (Google form) among CVD patients and the general Lebanese population. Eligible patients were ≥18-year-old, Lebanese CVD patient or non-CVD subjects from the general population, residing in Lebanon, and able to understand Arabic or English.

The population was targeted in all the eight governorates (Mohafazat) in Lebanon: Akkar, North, Beirut, Mount Lebanon, Bekaa, Baalbeck-Hermel, Nabatiyeh, and South. However, since the population is unequally distributed, we decided to regroup them into five governorates: Beirut, Mount Lebanon, Bekaa (Bekaa and Baalbeck-Hermel), North of Lebanon (North and Akkar), and South of Lebanon (South and Nabatieh).

### 3. Study population

The representative sample size of subjects with CVD was calculated using the Cochran formula n = {\textstyle{{{Z^2}pq} \over {{e^2}}}} , where Z^2^ is the square of the confidence interval considered, 95% in this case, which corresponds to (1.96)^2^, p is the estimated proportion of the Lebanese population which has CVD, q is (1-p), and e represents the p-value used which was set at 0.05. Therefore, a minimum of 355 patients suffering from CVD were required to participate in the study as a representative sample of the Lebanese population, based on the prevalence of CVD worldwide (36%) [7]. As for the group that included the general Lebanese population, a target of a minimum of 355 non-CVD subjects were required to fill out the questionnaire in order to be representative of the Lebanese general population. Data from 355 CVD patients and 355 non-CVD subjects (an average of 50 persons from 8 governates) was needed for analysis. Accordingly, 951 subjects were enrolled.

### 4. Procedures of data collection measurements

#### 4.1. Data sources

Data was collected using a pre-validated structured questionnaire ]. It was uploaded on Google Form. Non-CVD participants were recruited from the general population across Lebanon, whereas CVD patients were recruited from dispensaries and private clinics, such as Srebta Health Center and Armenian Relief Cross Lebanon Center. Most of our CVD patients and non-CVD subjects (95%) were directly interviewed via face-to-face interaction or over phone calls. The questionnaire required no more than 10 minutes to be filled and was available in both English and Arabic languages. The questionnaire was translated from English to Arabic language using the inverted method of Fortin [32]. The authors first translated it from English to Arabic. Then, the Arabic version was translated into English by a healthcare professional/translator to compare the agreement of the instrument. A pre-test was carried out with ten persons who were not part of the sample to validate the understanding and clarity of the questionnaire items. At the end of the pre-test, the questionnaire was modified as necessary [32].

#### 4.2. Variables

The questionnaire consisted of the following sections:

– Sociodemographic and other patient’s related characteristics: this section included 10 questions concerning the patient’s gender, age group, occupation, marital status, residency, education, personal monthly income, smoking status, alcohol drinking, presence of CVD, presence of medical illnesses, source of information about CVD.
– Knowledge about CVD: this section included 40 questions assessing how knowledgeable the patients are about CVD and their consequences of CVD, symptoms of coronary heart disease (CHD), risk factors of CVD, CVD risk levels (desirable values of high-density lipoprotein cholesterol (HDL-c), low-density lipoprotein cholesterol (LDL-c), fasting glycemia, normal blood pressure (BP) ranges, Normal BMI).
– Attitude and Practice toward CVD: These sections were comprised of 17 and 12 questions, respectively related to attitude and practice covering regular measuring of lipid profile, glycemia, and BP, diet plan, salt consumption, adherence to treatment, maintenance of normal body weight, and exercise.
– For KAP assessment, the widely adopted Bloom’s cutoff points are the following: 80–100% (good KAP), 60–79% (moderate KAP), and less than 60% (poor KAP) [33,34,35]. In this study, we used the Median of the scores and a modified Bloom’s cutoff values with the subcategories of ‘Poor’ and ‘Fair’ scores grouped under the category ‘limited KAP’ about CVD and subcategories of ‘Good’ and ‘Excellent’ scores grouped under the category of ‘adequate KAP’ about CVD. These cutoff values were also based on previously published KAP studies [36,37].

Computed scores were graded into categories and subcategories, as shown in Table 1.

**Table 1:** Grading of Knowledge (K), Attitude (A), and Practice (P) scores about CVD into Categories ‘Limited and Adequate’ and Sub-Categories ‘’Poor, Fair, Good, and Excellent’.


CATEGORIES	SUB-CATEGORIES	KNOWLEDGE		ATTITUDE		PRACTICE	
		
		/40	%	/85	%	/9	%

• **LIMITED**	• POOR	≤26	≤65	≤55	≤64.7	≤6	≤66.66

	• FAIR	[27,28,29,30,31,32]	[67.5–80]	[56–69]	[65.88–81.17]	7	77.77

• **ADEQUATE**	• GOOD	[33,34,35,36,37,38]	[82.5–95]	[70–82]	[82.8–97.1]	9	88.88

	• EXCELLENT	[39,40]	[97.5–100]	[83–85]	[97.64–100]	9	100


### 5. Data analysis

Collected data was inserted and analyzed using Statistical Package for Social Sciences (SPSS) software (version 25). The cumulative replies to each question were reported along with their respective percentages. Data were represented as frequencies and proportions for the nominal variables and as mean (±SD) for the continuous variables. Scores of KAP were computed. As such, 40 items were included for knowledge score, 17 for attitude score, and 9 for practice score. Sections of the knowledge and practices were scored in such a way that every correct answer was granted 1 point and each wrong answer a 0. Concerning the attitude section, a 5-point Likert scale was adopted in which ‘strongly disagree’ was given 1 point and ‘strongly agree’ was given 5 points for all items. The overall KAP score was calculated from the sum of the points granted where the cut-off value was the median for each section.

Descriptive analysis was used for the representation of the sample characteristics and the KAP data. Normality distribution was checked via the data representation on histograms and QQ plots and showed that the three scores were normally distributed. KAP scores and continuous variables were represented by mean, standard deviation, minimum and maximum. Categorical variables will be presented by their frequency and percentage. Bivariate analysis was conducted in order to test the correlation between the KAP scores and the demographic characteristics in the two study groups (CVD and non-CVD). Tests used were Student t-test and ANOVA test. In addition, the correlation was tested between the three KAP scores using Pearson correlation test. A multivariate analysis was enrolled in order to test factors affecting each of the three scores in the population. Significance level was set at 5%.

## Results

### 1. Demographic characteristics

A total of 921 participants filled our questionnaire form and were distributed over different governorates. Most of CVD patients were from Mount Lebanon (47.6%). A proportion of 52.6% of CVD patients were males, while non-CVD participants were 47.7% of males. No statistically significant differences were observed between groups in term of gender. The mean age of the participants was 47.3 ± 18.3 years old. CVD patients (58.3 ± 13.7 years old) were significantly older than non-CVD participants (36.3 ± 15.4 years old) (p < 0.001). A significantly greater proportion of CVD patients were married compared to non-CVD subjects, (77.6% vs 47.7%, respectively), while other CVD patients were widowed (11.1%), single (8.5%), and divorced (2.8%), p < 0.001.

Smoking was prevalent in 36.7% of our total population. Smoking has been found associated with CVD with a p value <0.001. For instance, 44.1% of CVD patients were smokers, while 55.9% were not. Conversely, only 29.3% of non-CVD participants were smokers while 70.7% were not (p < 0.001). In term of medical history, diabetes mellitus, hypercholesterolemia, obesity thyroid disease, and stroke were found associated with CVD with a p value <0.001. Indeed, CVD patients had a higher prevalence of diabetes compared to non-CVD participants (32.4% vs 5.9%), as well as of hypercholesterolemia (48.7% vs 7.6%), obesity (28.5% vs 7.8%), thyroid disease (13.9% vs 4.8%), and stroke occurrence (6.5% vs 0.4%). Other details about demographics can be found in Table 2.

**Table 2:** Bivariate analysis of demographics and other characteristics of the enrolled subjects (CVD patients vs Non-CVD participants) (N = 921).


		STUDY GROUPS		TOTAL	P.VALUE

		NON-CVD	CVD		

Governorate	Beirut	73	80	153	**<0.001**

		15.8%	17.4%	16.6%	

	North Lebanon	89	43	132	

		19.3%	9.3%	14.3%	

	South Lebanon	92	75	167	

		20.0%	16.3%	18.1%	

	Mount Lebanon	105	219	324	

		22.8%	47.6%	35.2%	

	Beqaa	102	43	145	

		22.1%	9.3%	15.7%	

Gender	Male	220	242	462	0.138

		47.7%	52.6%	50.2%	

	Female	241	218	459	

		52.3%	47.4%	49.8%	

Age	<45	318	60	378	**<0.001**

		69.0%	13.0%	41.0%	

	45 – 65	118	253	371	

		25.6%	55.0%	40.3%	

	>65	25	147	172	

		5.4%	32.0%	18.7%	

Age	Mean (SD)	36.3 (15.4)	58.3 (13.7)	47.3 (18.3)	**<0.001**

	Min – Max	18–86	19–90	18–90	

Marital status	Single	219	39	258	**<0.001**

		47.5%	8.5%	28.0%	

	Married	220	357	577	

		47.7%	77.6%	62.6%	

	Divorced	10	13	23	

		2.2%	2.8%	2.5%	

	Widowed	12	51	63	

		2.6%	11.1%	6.8%	

Occupation	Not working	136	86	222	**<0.001**

		29.5%	18.7%	24.1%	

	Working as healthcare professional	70	24	94	

		15.2%	5.2%	10.2%	

	Working as non-healthcare professional	192	197	389	

		41.6%	42.8%	42.2%	

	Household	63	153	216	

		13.7%	33.3%	23.5%	

Educational level	No formal education	12	24	36	**<0.001**

		2.6%	5.2%	3.9%	

	Elementary class	11	93	104	

		2.4%	20.2%	11.3%	

	Complementary	35	91	126	

		7.6%	19.8%	13.7%	

	Secondary	71	98	169	

		15.4%	21.3%	18.3%	

	University level	266	118	384	

		57.7%	25.7%	41.7%	

	Postgraduate	66	36	102	

		14.3%	7.8%	11.1%	

Personal Monthly income	0–750,000LL	157	124	281	**<0.001**

		34.1%	27.0%	30.5%	

	751,000–1,500,000LL	116	91	207	

		25.2%	19.8%	22.5%	

	1,501,000–3,000,000LL	97	91	188	

		21.0%	19.8%	20.4%	

	3,001,000–4,500,000LL	39	59	98	

		8.5%	12.8%	10.6%	

	More than 4,500,000LL	52	95	147	

		11.3%	20.7%	16.0%	

Smoking (Are you a current smoker?)	No	326	257	583	**<0.001**

		70.7%	55.9%	63.3%	

	Yes	135	203	338	

		29.3%	44.1%	36.7%	

Medical History	Diabetes mellitus	27	149	176	**<0.001**

		5.9%	32.4%	19.1%	

	High Cholesterol	35	224	259	**<0.001**

		7.6%	48.7%	28.1%	

	Obese	36	131	167	**<0.001**

		7.8%	28.5%	18.1%	

	Thyroid disease	22	64	86	**<0.001**

		4.8%	13.9%	9.3%	

	Stroke	2	30	32	**<0.001**

		0.4%	6.5%	3.5%	

Source of Information	Healthcare worker	221	353	574	**<0.001**

		47.9%	76.7%	62.3%	

	TV and radio	300	284	584	0.293

		65.1%	61.7%	63.4%	

	Newspapers or general magazines	128	97	225	**0.018**

		27.8%	21.1%	24.4%	

	Specialized health journals	177	72	249	**<0.001**

		38.4%	15.7%	27.0%	

	By watching medical conferences	132	42	174	**<0.001**

		28.6%	9.1%	18.9%	

	Through the experience of cardiac patient	219	294	513	**<0.01**


### 2. CVD-Related KAP

#### 2.1. KAP Scores about CVD according to categories and subcategories

Results showed that the 921 participants (CVD group and non-CVD) had an overall limited knowledge, attitude, and practice toward CVD. Specifically, the participants showed poor level of knowledge (25.05 ± 6.84 over 40 [62.62%]), fair level of attitude (62.34 ± 11.40 over 85 [73.34%]), and poor to fair levels of practice (6.15 ± 1.99 over 9 [68.33%]) concerning CVD. ‘Limited’ knowledge, attitude, and practice about CVD were reported in 89.9%, 70.5%, and 71.1% of the participants (n = 921), respectively (Table 3).

**Table 3:** Bivariate analysis of KAP scores about CVD and participants distribution (N) (CVD vs Non-CVD) according to subcategories (Poor, Fair, Good, Excellent).


		STUDY GROUPS		TOTAL	P.VALUE

		NON-CVD	CVD		

Knowledge (Subjects’ distribution)	Poor	283	202	485	**<0.001**

		61.4%	43.9%	52.7%	

	Fair	132	211	343	

		28.6%	45.9%	37.2%	

	Good	43	46	89	

		9.3%	10.0%	9.7%	

	Excellent	3	1	4	

		0.7%	0.2%	0.4%	

Knowledge score	Mean (SD)	23.5 (7.9)	26.6 (5.2)	25.0 (6.8)	**<0.001**

	Min – Max	0 – 40	5–39	0–40	

	% Mean over 40	58.75%	66.50%	62.50%	

Attitude (Subjects’ distribution)	Poor	140	98	238	**0.006**

		30.4%	21.3%	25.8%	

	Fair	188	224	412	

		40.8%	48.7%	44.7%	

	Good	133	136	269	

		28.9%	29.6%	29.2%	

	Excellent	0	2	2	

		0.0%	0.4%	0.2%	

Attitude score	Mean (SD)	61.4 (12.4)	63.3 (10.2)	62.3 (11.4)	**<0.001**

	Min – Max	21–82	29 – 83	21–83	

	% Mean over 85	72.23%	74.47%	73.29%	

Practice (Subjects’ distribution)	Poor	224	267	491	**<0.001**

		48.6%	58.0%	53.3%	

	Fair	63	101	164	

		13.7%	22.0%	17.8%	

	Good	84	60	144	

		18.2%	13.0%	15.6%	

	Excellent	90	32	122	

		19.5%	7.0%	13.2%	

Practice score	Mean (SD)	6.3 (2.2)	6.0 (1.7)	6.1 (2.0)	**<0.001**

	Min – Max	1–9	2–9	1–9	

	% Mean over 9	70.00%	66.66%	67.77%	


**Figure 1:**
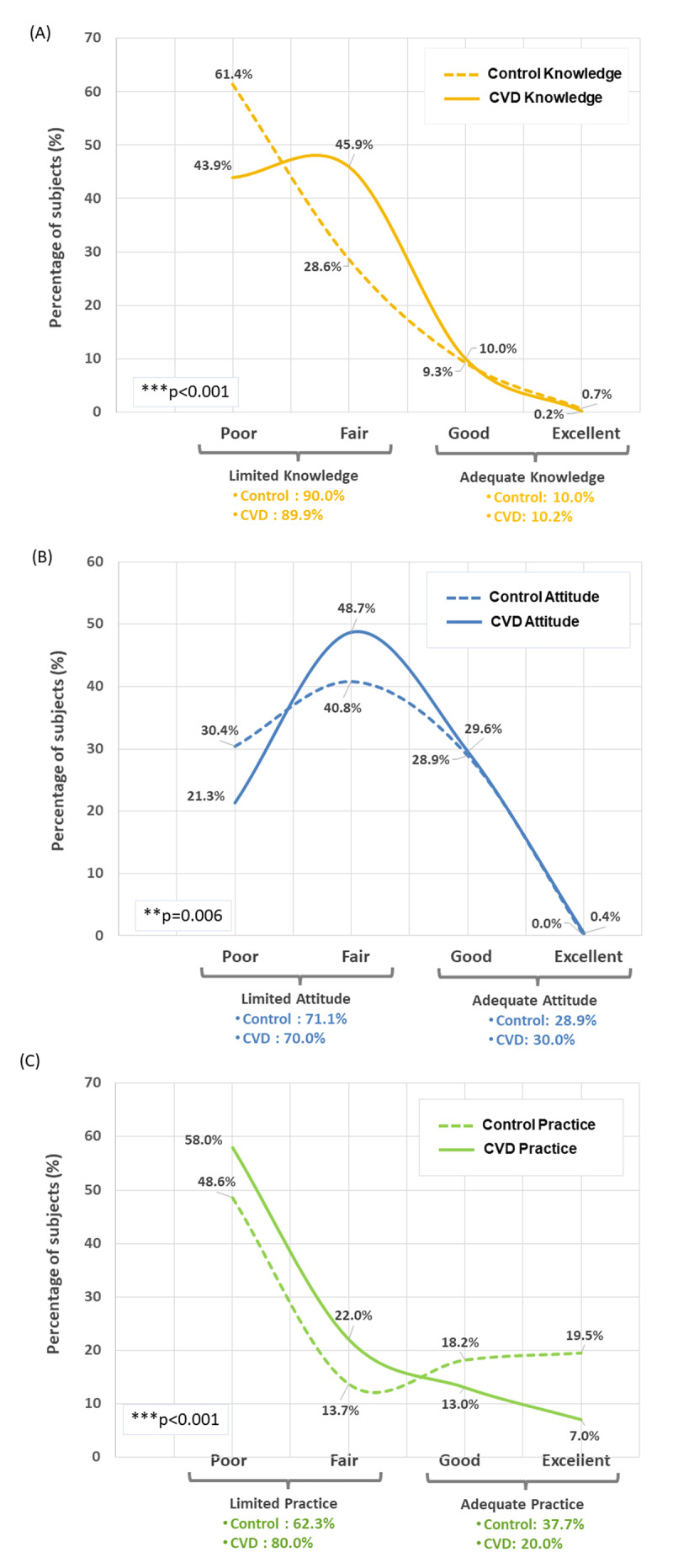
Percentage (%) of CVD patients versus Non-CVD subjects with Knowledge (A), Attitude (A), and Practice (P) scores represented in categories (Limited/Adequate) and sub-categories (Poor/Fair/Good/Excellent).

When each sub-population was analyzed separately, the mean CVD knowledge score in CVD patients was significantly higher than the one of non-CVD subjects (26.6 ± 5.2 over 40 [66.50%] poor to fair level vs 23.5 ± 7.9 over 40 [58.75%] poor level, respectively, p < 0.001) (Table 3 and Figure 1A). In addition, the mean CVD attitude score in CVD patients was significantly higher than the one of non-CVD subjects (63.3 ± 10.2 over 85 [74.47%] fair level vs 61.4 ± 12.4 over 85 (72.74%) fair level, respectively, p < 0.001) (Table 3 and Figure 1B). Furthermore, the mean CVD practice score in CVD patients was significantly lower than the one of non-CVD subjects (6.0 ± 1.7 over 9 [66.67%] poor to fair level vs 6.3 ± 2.2 over 9 [70.00%] poor to fair level, respectively, p < 0.001) (Table 3 and Figure 1C).

#### 2.2. CVD-Related Knowledge

Among all participants in the study, 45.9% correctly recognized that CVD are the leading cause of death in Lebanon, 48.4% knew that most CVD cases are hereditary [38], and only 35.5% were aware that CVD are the primary cause of death in diabetic patients. Moreover, 91% knew that hypertension is a CVD risk factor, and 32% that cancer are CVD risk factors. Detailed responses to the knowledge section are provided in Table S1.

#### 2.3. CVD-Related Attitude

Attitude towards CVD included 17 items following a Likert scale from 1 (strongly disagree) to 5 (strongly agree). The top three attitude items for which participants showed the best scores were ‘Take treatment as recommended by doctor’ (4.9 ± 1.16 over 5 [98%]), ‘Should know blood pressure level’ (4.12 ± 1.22 over 5 [82.4%]), and ‘Should know blood sugar level’ (4.0 ± 1.27 over 5 [80%]). On the other hand, the bottom three items for which participants showed the worst scores were ‘Willingness to take hormone replacement therapy (HRT)’ (2.11 ± 1.28 over 5 [42.2%]), ‘Eat with restriction as feel well’ (3.19 ± 1.47 over 5 [63.8%]), ‘Change eating habit easily’ (3.39 ± 1.42 over 5 [67.8%]), Attitudes items are set in Table S2.

#### 2.4. CVD-Related Practice

Among all participants, 55.4% performed exercise more than 20 min 3x/week, 75.7% were taking fatty food more than three times/week, 81% maintained normal weight, 63.5% tried to reduce stress, 65.4% were nor smokers and used to be passive smokers, 91.9% took treatment as recommended by doctor, 69.4% visited doctor for advice regularly, only 38.4% took omega3 for heart disease prevention, and 73.9% increased knowledge about CVD through mass media or internet, as shown in Table S3.

### 3. Correlation among kap score parameters

Results of the Pearson correlation test revealed that knowledge in the non-CVD group was positively correlated with attitude (p-value < 0.001, r = 0.182) and practice (p-value = 0.011, r = 0.118). In addition, attitude was positively correlated with practice (p-value < 0.001, r = 0.332).

As for the CVD group, results of the Pearson correlation test revealed that knowledge was positively correlated with attitude (p-value < 0.001, r = 0.312) and practice (p-value <0.001, r = 0.244). In addition, attitude was positively correlated with practice (p-value < 0.001, r = 0.441).

### 4. Socio-demographiic characteristics of cvd patients & Non-cvd participants with poor knoweldge, poor attitude, and poor practice scores

Percentages of Lebanese CVD patients and non-CVD subjects with Poor Knowledge score (K: ≤26 over 40 so ≤65.0%), Poor Attitude score (A: ≤55 over 85 so ≤64.7%), and Poor Practice score (P: ≤6 over 9 so ≤66.66%) regarding CVD according to socio-demographic status are represented in Table 4.

**Table 4:** Percentage (%) of Lebanese CVD patients and non-CVD subjects (Control) with Poor Knowledge, Poor Attitude, and Poor Practice regarding CVD according to socio-demographic status.


			% POOR KNOWLEDGE	% POOR ATTITUDE	% POOR PRACTICE

**GENDER**	**MALE**	NON-CVDNon-CVD	**60.0%**	35.9%	**59.5%**

		CVD	42.1%	21.9%	**57.0%**

	**FEMALE**	NON-CVDNon-CVD	**62.7%**	25.3%	38.6%

		CVD	45.9%	20.6%	**59.2%**

**AGE**	**<45**	NON-CVDNon-CVD	**57.2%**	27.7%	46.9%

		CVD	45.0%	16.7%	**55.0%**

	**45–65**	NON-CVD	**71.2%**	34.7%	**55.1%**

		CVD	40.7%	28.6%	**56.5%**

	**>65**	NON-CVD	**68.0%**	44.0%	40.0%

		CVD	**49.0%**	21.3%	**61.9%**

**MARITAL STATUS**	**SINGLE**	NON-CVD	**53.0%**	26.0%	44.3%

		CVD	35.9%	23.1%	46.2%

	**MARRIED**	NON-CVD	**67.7%**	34.1%	**51.8%**

		CVD	42.3%	20.2%	**57.1%**

	**DIVORCED**	NON-CVD	**70.0%**	20.0%	**60.0%**

		CVD	38.5%	15.4%	**69.2%**

	**WIDOWED**	NON-CVD	**91.7%**	**50.0%**	**58.3%**

		CVD	**62.7%**	29.4%	**70.6%**

**EDUCATION LEVEL**	**NO FORMAL EDUCATION**	NON-CVD	**83.3%**	**66.7%**	**50.0%**

		CVD	**66.7%**	33.3%	**58.3%**

	**ELEMENTARY CLASS**	NON-CVD	**72.7%**	**54.5%**	**72.7%**

		CVD	**61.3%**	30.1%	**73.1%**

	**COMPLEMENTARY**	NON-CVD	**65.7%**	**54.3%**	**54.3%**

		CVD	45.1%	22.0%	**54.9%**

	**SECONDARY**	NON-CVD	**74.6%**	33.8%	**54.9%**

		CVD	36.7%	15.3%	**49.0%**

	**UNIVERSITY LEVEL**	NON-CVD	**56.8%**	26.7%	48.1%

		CVD	35.6%	16.9%	**58.5%**

	**POSTGRADUATE**	NON-CVD	**57.6%**	18.2%	36.4%

		CVD	27.8%	19.4%	**50.0%**

**OCCUPATION**	**NOT WORKING**	NON-CVD	**64.7%**	28.7%	**52.9%**

		CVD	45.3%	26.7%	**58.1%**

	**WORKING AS HEALTHCARE PROFESSIONAL**	NON-CVD	20.0%	20.0%	32.9%

		CVD	8.3%	25.0%	**58.3%**

	**WORKING AS NON-HEALTHCARE PROFESSIONAL**	NON-CVD	**72.9%**	35.4%	**54.2%**

		CVD	46.7%	18.8%	**55.8%**

	**HOUSEHOLD**	NON-CVD	**65.1%**	30.2%	39.7%

		CVD	45.1%	20.9%	**60.8%**

**GOVERNORATES**	**BEIRUT**	NON-CVD	46.6%	26.0%	39.7%

		CVD	35.0%	15.0%	**51.3%**

	**NORTH LEBANON**	NON-CVD	**62.9%**	30.3%	**51.7%**

		CVD	**55.8%**	11.6%	**65.1%**

	**SOUTH** **LEBANON**	NON-CVD	**63.0%**	25.0%	43.5%

		CVD	**56.0%**	21.3%	**62.7%**

	**MOUNT LEBANON**	NON-CVD	**64.8%**	30.5%	**54.3%**

		CVD	40.6%	26.9%	**62.1%**

	**BEQAA**	NON-CVD	**65.7%**	38.2%	**51.0%**

		CVD	44.2%	14.0%	34.9%

**PERSONAL INCOME**	**0–750,000LL**	NON-CVD	**65.6%**	29.3%	**53.5%**

		CVD	46.0%	23.4%	**58.9%**

	**751,000–1,500,000LL**	NON-CVD	**64.7%**	42.2%	46.6%

		CVD	**50.5%**	23.1%	**56.0%**

	**1,501,000–3,000,000LL**	NON-CVD	**63.9%**	27.8%	47.4%

		CVD	41.8%	17.6%	**56.0%**

	**3,001,000–4,500,000LL**	NON-CVD	**64.1%**	30.8%	35.9%

		CVD	35.6%	23.7%	**55.9%**

	**More than 4,500,000LL**	NON-CVD	34.6%	11.5%	**50.0%**

		CVD	42.1%	18.9%	**62.1%**

**SMOKING STATUS**	**NO**	NON-CVD	**57.1%**	24.8%	42.6%

		CVD	37.7%	16.7%	46.7%

	**YES**	NON-CVD	**71.9%**	43.7%	**63.0%**

		CVD	**51.7%**	27.1%	**72.4%**


In all socio-demographic characteristics, a larger number of non-CVD subjects when compared to CVD patients recorded a poorer CVD knowledge score and CVD attitude score. For instance, 72.9% of non-CVD non-healthcare workers and 71.9% of non-CVD subjects smokers vs 46.7% of CVD patients non-healthcare workers and 51.7% of CVD patients smokers, respectively, had a poor CVD knowledge score. Moreover, 66.7% of non-CVD subjects with no formal education vs 33.3% of CVD patients with no formal education had a poor CVD attitude score (Table 4).

Conversely, in almost all socio-demographic characteristics, a larger number of CVD patients when compared to non-CVD subjects recorded a poorer CVD practice score. For instance, 72.4% of smokers among CVD patients vs 63.0% of smokers among non-CVD subjects had a poor CVD practice score (Table 4).

### 5. Factors affecting kap scores of the enrolled cvd patients & Non-cvd participants

Bivariate analysis was used to identify factors affecting the knowledge, attitude and practice scores in both populations CVD patients and non-CVD participants.

The knowledge score in CVD patients was significantly associated with age, marital status, healthcare workers, area of residency, educational level, and smoking status. In non-CVD participants, the knowledge score was associated with the same factors apart the age of participants. In addition, non-CVD participants with high monthly income knew significantly more about CVD. More details are presented in Table S4.

The Attitudes score in CVD patients was significantly associated with age, area of residency, educational level, and smoking status (p < 0.001). In non-CVD participants, the attitude score was associated with gender, working status, educational level, monthly income, and smoking status. More details are presented in Table S5.

Finally, the Practice score in CVD patients was associated with area of residency, and educational level. As for the practice score in non-CVD participants, it was significantly associated with gender, working status, area of residency, educational level, and smoking status. More details are presented in Table S6.

### 6. Predictors of kap among cvd patients & Non-cvd participants

Multiple linear regression was applied to identify predictors of knowledge among CVD patients & non-CVD participants.

In CVD patients, knowledge about CVD was significantly (p < 0.05) and positively associated with the educational level, the healthcare profession, the living area in Beirut, but negatively associated with smoking, and the living area in South Lebanon. In non-CVD participants, knowledge about CVD was significantly (p < 0.05) and positively associated with the healthcare profession, the living area in Beirut, but negatively associated with smoking, and the widow status (Table S7).

In CVD patients, attitude towards CVD was positively associated with the knowledge about CVD and educational level, but negatively associated with smoking and the living area in Mount Lebanon. In non-CVD participants, Attitude towards CVD was associated with same predictors than those in CVD patients apart the living area in Mount Lebanon (Table S7).

In CVD patients, practice towards CVD was positively associated with the attitudes towards CVD, the living area in beqaa, but negatively associated with smoking, and the widow status. On the other hand, in non-CVD participants, practice toward CVD was positively associated with the attitudes towards about CVD, the healthcare profession, female gender, but negatively associated with smoking, and the living area in Mount Lebanon (Table S7).

## Discussion

The World Health Organization classifies CVDs among the leading causes of mortality worldwide [2,39]. Our KAP study targeted 460 CVD patients and 461 non-CVD participants, and both groups included an equal gender distribution. All considered risk factors were found to be more prevalent among CVD patients than non-CVD subjects, which stands in accordance with historical perspectives on CVD risk indicators [40]. When considering the whole sample of population in our study, our participants showed an overall poor level of knowledge, a fair level of attitude, and poor to fair levels of practice toward CVD.

**Figure 2:**
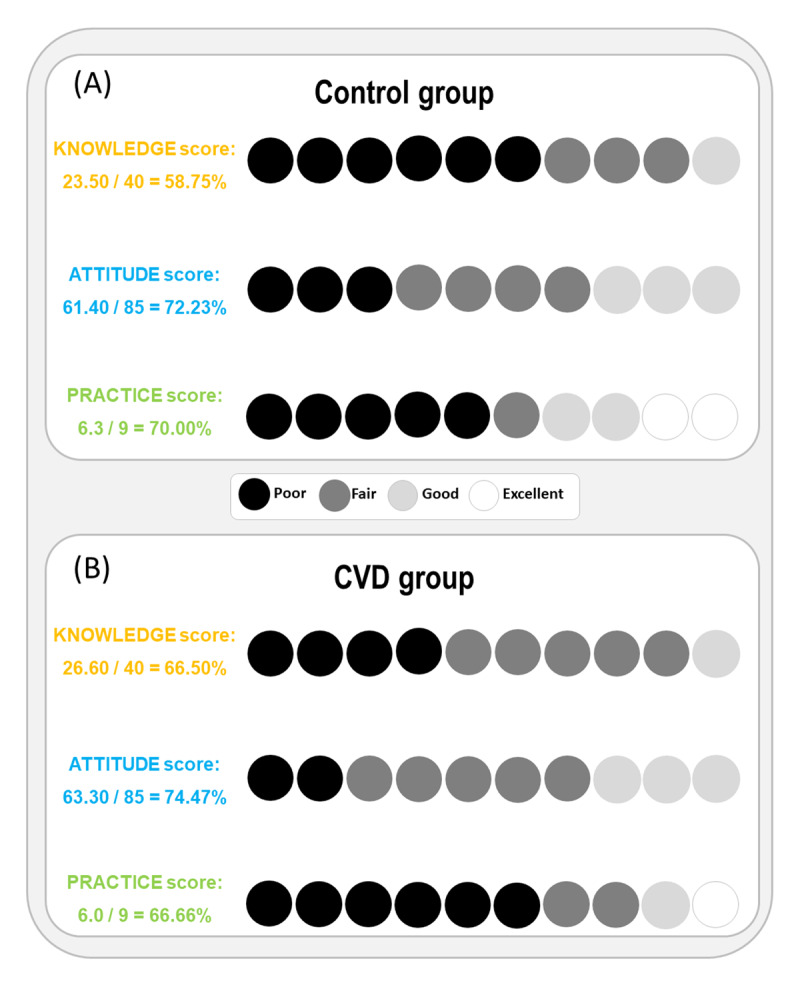
Knowledge (K), attitude (A), and practice (P) scores in Non-CVD subjects (A) vs CVD patients (B). Schematic representation of the Percentage (%) of CVD patients versus Control subjects with KAP scores represented in sub-categories (Poor/Fair/Good/Excellent). Each circle represents approximately 10% of the participants.

Considering each group separately (CVD vs non-CVD), CVD patients showed more knowledge about CVD than the non-CVD participants, but the knowledge level in both sub-populations remained limited (Figures 1 & 2), as was also reported in other studies. For instance, Wang et al. found that both CVD and non-CVD groups had a lower level of knowledge before receiving rehabilitation education and health education, respectively [41]. Waśniowska et al. showed that knowledge of cardiovascular risk factors was very insufficient in residents of Małopolska Voivodeship [42]. Similarly, Rosediani et al. concluded that only a minority of women in North-Eastcoast Malaysia were aware of atypical CVD symptoms such as nausea, jaw and left shoulder pain [43]. However, both Nursyafiza et al. and Koohi et al. studies, conducted in Kuantan and Tehran respectively, showed that participants had satisfactory knowledge related to CVDs and their risk factors [27,29]. In our study, the majority knew that CVDs affect both men and women and people of all ages. However, less than 50% knew that CVD is the leading cause of death in their home country, which was similar to the results of Koniak-Griffin and Brecht among Latina women [26]. Although patients in their study were from distinct countries, this result might be due to a breach in awareness of CVDs in third world countries, as suggested in other studies [44,45]. Most patients in our study recognized hypertension and diabetes mellitus as CVD risk factors. Similarly, other studies showed that patients were mainly aware of diabetes, hypertension, and smoking as risk factors for CVD [29,34,46]. However, the majority of our participants were unaware about cancer, asthma, and allergies as CVD factors.

CVD patients showed better attitude toward CVD than the non-CVD subjects but attitude levels in both groups also remained within fair levels. In Wang et al.’s study, the CVD group reached 93.94% positive attitude, which was much higher than the non-CVD participants [41]. In contrast to our study, Koohi and Khalili had satisfactory levels of attitude among their participants [29]. In our study, as for attitudes, results showed that the majority of patients had the willingness to take on healthier patterns, such as regular exercise, healthier eating habits, and take treatments as directed.

CVD patients had worse practice toward CVD than the non-CVD subjects but practice levels in both groups remained low. The overall score of the ‘Behavior’ dimension (practice component) was higher in the CVD group than in the non-CVD group in Wang et al.’s study [41]. Some good practices were found among our participants, such as performing regular exercise, maintaining a normal weight, reducing stress, and being non-smokers. Nevertheless, some behaviors were negative such as regular fatty food intake, and only a low percentage took omega 3 supplements for heart attack prevention. The lack of omega 3 intake is consistent with a study conducted in Kelantan where only 15.8% of patients took this supplement for CVD prevention [47]. This can be due to the lack of awareness of the benefits of this supplement when it comes to CVDs.

Analysis on KAP in CVD patients and non-CVD subjects found that educational level, healthcare profession, and living in Beirut (urban area) were predictors of better CVD knowledge in both groups. In compliance with Aminde’s study findings, high education levels and higher income implied a better knowledge of CVDs, however, in their study, former smokers had a higher knowledge level than non-smokers [30]. CVD knowledge, educational level and being a non-smoker were predictors of better attitudes in both sub-populations as well. Finally, CVD attitudes and being a non-smoker were predictors of better practices in both CVD and non-CVD patients, in addition to female gender and healthcare profession as predictors of good practice in the non-CVD group only. Our results were close to those of Vaidya et al. which showed a gap between CVD knowledge, attitude and practice in a semiurban community in a low-income nation, even among those already affected by CVD [48]. Finally, it is worth noting that there was a statistically significant age gap of 22 years between CVD and non-CVD groups, with CVD patients being more likely middle-aged and older adults. This might have influenced several of our findings. Particularly, CVD patients were more likely to have smoked, have lower educational levels, and to depend on their healthcare provider for information regarding CVD. This could explain the limited awareness level among this group. On the other hand, non-CVD participants were less likely to suffer from any chronic disease, thus they were less concerned about CVD related knowledge and practice.

Our findings showed that better knowledge and good attitudes lead to better self-reported practices. This can be further promoted through the implementation of strategies, and through encouragement to comply with prevention and treatment practices to better control CVD and bring core behavioral change to people prone to or suffering from CVD.

## Limitations

The Coronavirus 2019 (COVID-19) pandemic has presented itself as a tremendous challenge in regard to data collection. Indeed, recruitment of subjects and face to face interviews were hindered because of the lockdown, curfews, and decrease in medical visits, ambulatory care, and hospital admissions. To overcome this, we recurred to phone call interviews. Furthermore, this study was subject to selection, volunteering, and reporting bias since the surveying method limited the ability to reach illiterate or underprivileged populations and it was a self-reported survey. However, to reduce these biases our team was well trained to address around 95% of the overall CVD patients and non-CVD subjects via face-to-face or phone interviews.

## Perspectives

**Figure 3:**
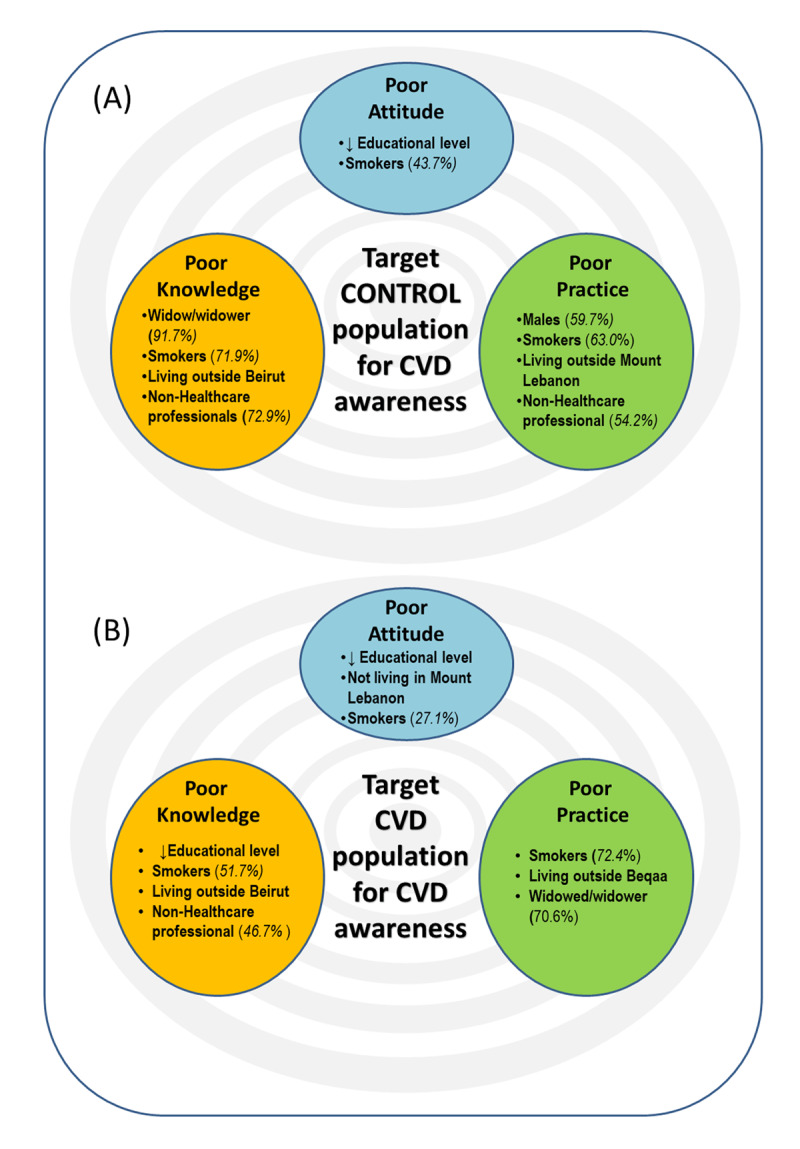
Target population for CVD awareness: Major factors associated with poor CVD knowledge, attitude, and practice in Non-CVD subjects (A) vs CVD patients (B). The % represents the percentage of subjects.

This is the most recent CVD KAP study in Lebanon involving CVD patients and non-CVD subjects residing in Lebanon from different backgrounds. Future studies should consider conducting large scale KAP surveys in order to better investigate the level of awareness of CVD among the Lebanese population. This study identified some predictors of KAP in CVD patients and non-CVD subjects thus allowing the screening of CVD patients or those with some predisposing risk factors. In addition, it showed limited knowledge, attitude, and practice toward CVD (Figure 3), thus national health authorities are urged to implement strategies and programs to improve the population’s KAP about CVDs, as this would prompt the community to seek appropriate preventative and remedial care. Also, it is important for the public health sector to work with the media to educate the Lebanese community on symptoms, risk factors, prevention methods and treatments of CVD.

## Conclusion

This study revealed that participants residing in Lebanon showed poor level of knowledge, fair level of attitude, and poor to fair levels of practice concerning CVD. Surprisingly, ‘Adequate’ (good+ excellent) knowledge, attitude, and practice about CVD were reported only in 10.1%, 29.5%, and 28.9% of the participants (n = 921), respectively. Our study also pinpointed predictors of CVD knowledge, attitudes, and practices among CVD and non-CVD participants, which allows the identification of vulnerable patients in order to target them in future awareness campaigns (Figure 3).

## Additional File

The additional file for this article can be found as follows:

10.5334/gh.1138.s1 Supplementary Tables. Tables S1 to S7.
