# Supplementary material for: Knowledge, Attitude, and Practice Toward Cardiovascular Diseases in the Lebanese Population
Source: Glob Heart. 2022 Jul 29;17(1):47. doi: 10.5334/gh.1138 (PMC9336688; doi:10.5334/gh.1138)
Supplement: Supplementary Tables. — Tables S1 to S7. [file gh-17-1-1138-s1.pdf]

Table S1: Distribution of patients according to their responses to knowledge items about CVD (N=921)

		Frequency	Percent	Answer
CVD is related to heart	TRUE	825	89.6	Correct
	FALSE	41	4.5	Incorrect
	I do not know	55	6.0	Incorrect
CVD is related to obstructed blood vessels	TRUE	789	85.7	Correct
	FALSE	37	4.0	Incorrect
	I do not know	95	10.3	Incorrect
CVD is the leading cause of death in Lebanon	TRUE	423	45.9	Correct
	FALSE	200	21.7	Incorrect
	I do not know	298	32.4	Incorrect
CVD is the disease of women only	TRUE	26	2.8	Incorrect
	FALSE	815	88.5	Correct
	I do not know	80	8.7	Incorrect
CVD occurs in young people only	TRUE	35	3.8	Incorrect
	FALSE	810	87.9	Correct
	I do not know	76	8.3	Incorrect
Most CVD cases are hereditary	TRUE	446	48.4	Correct
	FALSE	246	26.7	Incorrect
	I do not know	229	24.9	Incorrect
Cardiovascular disease is the primary cause of death in diabetic patients	TRUE	327	35.5	Correct
	FALSE	194	21.1	Incorrect
	I do not know	400	43.4	Incorrect
Smoking is a CVD risk factor	TRUE	862	93.6	Correct
	FALSE	33	3.6	Incorrect
	I do not know	26	2.8	Incorrect
Regular exercise reduces CVD risk	TRUE	802	87.1	Correct
	FALSE	73	7.9	Incorrect
	I do not know	46	5.0	Incorrect
High LDL cholesterol is a CVD risk factor	TRUE	817	88.7	Correct
	FALSE	31	3.4	Incorrect
	I do not know	73	7.9	Incorrect
Stress reduces CVD risk	TRUE	237	25.7	Incorrect
	FALSE	550	59.7	Correct
	I do not know	134	14.5	Incorrect
High blood pressure is a CVD risk factor	TRUE	818	88.8	Correct
	FALSE	28	3.0	Incorrect
	I do not know	75	8.1	Incorrect
Family history of heart disease is a CVD risk factor	TRUE	753	81.8	Correct
	FALSE	66	7.2	Incorrect

	I do not know	102	11.1	Incorrect
History of previous heart attack is a CVD risk factor	TRUE	714	77.5	Correct
	FALSE	49	5.3	Incorrect
	I do not know	158	17.2	Incorrect
Increasing age (>55years IN women and >45 in men) is a CVD risk factor	TRUE	629	68.3	Correct
	FALSE	122	13.2	Incorrect
	I do not know	170	18.5	Incorrect
Obesity reduces CVD risk	TRUE	176	19.1	Incorrect
	FALSE	694	75.4	Correct
	I do not know	51	5.5	Incorrect
Light walking can prevent CVD	TRUE	622	67.5	Correct
	FALSE	177	19.2	Incorrect
	I do not know	122	13.2	Incorrect
Fruit and/or vegetables prevent CVD	TRUE	522	56.7	Correct
	FALSE	209	22.7	Incorrect
	I do not know	190	20.6	Incorrect
Adequate exercise prevents CVD	TRUE	677	73.5	Correct
	FALSE	159	17.3	Incorrect
	I do not know	85	9.2	Incorrect
Smoking is a CVD risk factor	TRUE	862	93.6	Correct
	FALSE	33	3.6	Incorrect
	I do not know	26	2.8	Incorrect
Regular exercise reduces CVD risk	TRUE	802	87.1	Correct
	FALSE	73	7.9	Incorrect
	I do not know	46	5.0	Incorrect
High LDL cholesterol is a CVD risk factor	TRUE	817	88.7	Correct
	FALSE	31	3.4	Incorrect
	I do not know	73	7.9	Incorrect
Stress reduces CVD risk	TRUE	237	25.7	Incorrect
	FALSE	550	59.7	Correct
	I do not know	134	14.5	Incorrect
High blood pressure is a CVD risk factor	TRUE	818	88.8	Correct
	FALSE	28	3.0	Incorrect
	I do not know	75	8.1	Incorrect
Family history of heart disease is a CVD risk factor	TRUE	753	81.8	Correct
	FALSE	66	7.2	Incorrect
	I do not know	102	11.1	Incorrect
History of previous heart attack is a CVD risk factor	TRUE	714	77.5	Correct
	FALSE	49	5.3	Incorrect
	I do not know	158	17.2	Incorrect
Increasing age (>55years IN women and >45 in men) is a CVD risk factor	TRUE	629	68.3	Correct
	FALSE	122	13.2	Incorrect
	I do not know	170	18.5	Incorrect

Obesity reduces CVD risk	TRUE	176	19.1	Incorrect
	FALSE	694	75.4	Correct
	I do not know	51	5.5	Incorrect
Light walking can prevent CVD	TRUE	622	67.5	Correct
	FALSE	177	19.2	Incorrect
	I do not know	122	13.2	Incorrect
Fruit and/or vegetables prevent CVD	TRUE	522	56.7	Correct
	FALSE	209	22.7	Incorrect
	I do not know	190	20.6	Incorrect
Adequate exercise prevents CVD	TRUE	677	73.5	Correct
	FALSE	159	17.3	Incorrect
	I do not know	85	9.2	Incorrect
Hypertension is a CVD risk factor	TRUE	838	91.0	Correct
	FALSE	18	2.0	Incorrect
	I do not know	65	7.1	Incorrect
Cancer is a CVD risk factor	TRUE	295	32.0	Correct
	FALSE	322	35.0	Incorrect
	I do not know	304	33.0	Incorrect
Diabetes Mellitus is a CVD risk factor	TRUE	657	71.3	Correct
	FALSE	96	10.4	Incorrect
	I do not know	168	18.2	Incorrect
Heart Attack is a CVD risk factor	TRUE	816	88.6	Correct
	FALSE	32	3.5	Incorrect
	I do not know	73	7.9	Incorrect
Asthma is a CVD risk factor	TRUE	312	33.9	Correct
	FALSE	304	33.0	Incorrect
	I do not know	305	33.1	Incorrect
Stroke is a CVD risk factor	TRUE	549	59.6	Correct
	FALSE	159	17.3	Incorrect
	I do not know	213	23.1	Incorrect
Allergies are CVD risk factor	TRUE	159	17.3	Correct
	FALSE	467	50.7	Incorrect
	I do not know	295	32.0	Incorrect
Chest pain is a CHD symptom	TRUE	762	82.7	Correct
	FALSE	42	4.6	Incorrect
	I do not know	117	12.7	Incorrect
Pain at the jaw, neck and left shoulder is a CHD symptom	TRUE	659	71.6	Correct
	FALSE	71	7.7	Incorrect
	I do not know	191	20.7	Incorrect
Sweating is a CHD symptom	TRUE	640	69.5	Correct
	FALSE	104	11.3	Incorrect
	I do not know	177	19.2	Incorrect
Palpitation is a CHD symptom	TRUE	649	70.5	Correct

	FALSE	66	7.2	Incorrect
	I do not know	206	22.4	Incorrect
Headache is a CHD symptom	TRUE	422	45.8	Correct
	FALSE	221	24.0	Incorrect
	I do not know	278	30.2	Incorrect
Epigastric pain (known as stomach ache) is a CHD symptom	TRUE	380	41.3	Correct
	FALSE	216	23.5	Incorrect
	I do not know	325	35.3	Incorrect
Shortness of breath is a CHD symptom	TRUE	728	79.0	Correct
	FALSE	61	6.6	Incorrect
	I do not know	132	14.3	Incorrect
Nausea and vomiting are CHD symptoms	TRUE	502	54.5	Correct
	FALSE	163	17.7	Incorrect
	I do not know	256	27.8	Incorrect
Dizziness is a CHD symptom	TRUE	450	48.9	Correct
	FALSE	180	19.5	Incorrect
	I do not know	291	31.6	Incorrect
A desirable level of HDL-C (Good cholesterol) level is:	< 40 mg/dl	55	6.0	Incorrect
	Between 40 and 60 mg/dl	224	24.3	Incorrect
	60 mg/dl or above	93	10.1	Correct
	I do not know	549	59.6	Incorrect
A desirable level of LDL-C (Bad cholesterol) is:	< 100 mg/dl	255	27.7	Correct
	Between 100 and 190 mg/dl	117	12.7	Incorrect
	190 mg/dl or above	16	1.7	Incorrect
	I do not know	533	57.9	Incorrect
Fasting blood sugar is considered normal if its value is:	70-100 mg/dl	479	52.0	Correct
	101-125 mg/dl	145	15.7	Incorrect
	126 mg/dl or above	6	0.7	Incorrect
	I do not know	291	31.6	Incorrect
Blood pressure is considered normal if its value is:	Systolic pressure between 90-119 and Diastolic pressure between 60-79	385	41.8	Incorrect
	Systolic pressure between 120-139 and Diastolic pressure between 80-89	309	33.6	Correct
	Systolic pressure between 140-159 and Diastolic pressure between 90-99	11	1.2	Incorrect
	I do not know	216	23.5	Incorrect

BMI (Body Mass Index) is considered normal if its value is:	18,5-24,9 kg/m2	225	24.4	Correct
	25-29,9 kg/m2	48	5.2	Incorrect
	30 kg/m2 or higher	9	1.0	Incorrect
	I do not know	639	69.4	Incorrect

Table S2: **Distribution of patients according to their responses to attitude items about CVD along with the respective mean score and standard deviation (N=921)**

		Answer	Frequency	Percent	Mean	SD
Willing to exercise	Strongly disagree	I	119	12.9	3.65	1.43
	Disagree	I	90	9.8		
	Neutral	I	171	18.6		
	Agree	I	153	16.6		
	Strongly agree	C	388	42.1		
Change eating habit easily	Strongly disagree	I	133	14.4	3.39	1.42
	Disagree	I	121	13.1		
	Neutral	I	214	23.2		
	Agree	I	160	17.4		
	Strongly agree	C	293	31.8		
Eat without restriction as feel well	Strongly disagree	C	181	19.7	3.19	1.47
	Disagree	I	123	13.4		
	Neutral	I	207	22.5		
	Agree	I	159	17.3		
	Strongly agree	I	251	27.3		
Maintain normal weight	Strongly disagree	I	67	7.3	3.93	1.23
	Disagree	I	49	5.3		
	Neutral	I	178	19.3		
	Agree	I	213	23.1		
	Strongly agree	C	414	45.0		
Enjoy life without healthy lifestyle	Strongly disagree	C	128	13.9	3.46	1.40
	Disagree	I	108	11.7		
	Neutral	I	193	21.0		
	Agree	I	193	21.0		
	Strongly agree	I	299	32.5		
Not smoking or being a passive smoker	Strongly disagree	I	186	20.2	3.58	1.60
	Disagree	I	75	8.1		
	Neutral	I	120	13.0		
	Agree	I	101	11.0		
	Strongly agree	C	439	47.7		
Not ready to change the lifestyle	Strongly disagree	C	130	14.1	3.52	1.41
	Disagree	I	89	9.7		
	Neutral	I	195	21.2		

	Agree	I	186	20.2		
	Strongly agree	I	321	34.9		
Willingness to take Hormone Replacement Therapy (HRT)	Strongly disagree	I	474	51.5	2.11	1.38
	Disagree	I	140	15.2		
	Neutral	I	137	14.9		
	Agree	I	74	8.0		
	Strongly agree	C	96	10.4		
Take treatment as recommended by doctor	Strongly disagree	I	54	5.9	4.29	1.16
	Disagree	I	37	4.0		
	Neutral	I	93	10.1		
	Agree	I	144	15.6		
	Strongly agree	C	593	64.4		
Do regular medical checkup	Strongly disagree	I	92	10.0	3.85	1.35
	Disagree	I	69	7.5		
	Neutral	I	157	17.0		
	Agree	I	167	18.1		
	Strongly agree	C	436	47.3		
Prefer traditional medicine such as acupuncture (fine needles inserted in the skin)/Chinese medicine	Strongly disagree	C	121	13.1	3.76	1.43
	Disagree	I	62	6.7		
	Neutral	I	165	17.9		
	Agree	I	145	15.7		
	Strongly agree	I	428	46.5		
Should know blood sugar level	Strongly disagree	I	71	7.7	4.00	1.27
	Disagree	I	58	6.3		
	Neutral	I	140	15.2		
	Agree	I	184	20.0		
	Strongly agree	C	468	50.8		
Should know blood pressure level	Strongly disagree	I	59	6.4	4.12	1.22
	Disagree	I	53	5.8		
	Neutral	I	120	13.0		
	Agree	I	177	19.2		
	Strongly agree	C	512	55.6		
Should know lipid level	Strongly disagree	I	69	7.5	3.98	1.25
	Disagree	I	56	6.1		
	Neutral	I	151	16.4		
	Agree	I	192	20.8		
	Strongly agree	C	453	49.2		
Try to reduce sugar intake	Strongly disagree	I	69	7.5	3.83	1.29
	Disagree	I	88	9.6		
	Neutral	I	183	19.9		
	Agree	I	170	18.5		

	Strongly agree	C	411	44.6		
Try to reduce fat intake	Strongly disagree	I	63	6.8	3.96	1.25
	Disagree	I	71	7.7		
	Neutral	I	148	16.1		
	Agree	I	199	21.6		
	Strongly agree	C	440	47.8		
Increase knowledge about CVD through mass media or electronic	Strongly disagree	I	114	12.4	3.72	1.39
	Disagree	I	69	7.5		
	Neutral	I	166	18.0		
	Agree	I	182	19.8		
	Strongly agree	C	390	42.3		

Table S3: Distribution of patients according to their responses to practice items about CVD included in the scoring system (N=921)

		Frequency	Percent	Answer
Exercise more than 20 min 3x/week	No	411	44.6	Incorrect
	Yes	510	55.4	Correct
Taking fatty food more than 3 times/week	No	697	75.7	Correct
	Yes	224	24.3	Incorrect
Maintain normal weight	No	175	19.0	Incorrect
	Yes	746	81.0	Correct
Reduce stress	No	336	36.5	Incorrect
	Yes	585	63.5	Correct
Not smoking or being a passive smoker	No	319	34.6	Incorrect
	Yes	602	65.4	Correct
Take treatment as recommended by doctor	No	75	8.1	Incorrect
	Yes	846	91.9	Correct
Visit doctor for advice regularly	No	282	30.6	Incorrect
	Yes	639	69.4	Correct
Taking omega3 for heart disease prevention	No	567	61.6	Incorrect
	Yes	354	38.4	Correct
Increase knowledge about CVD through mass media or internet	No	240	26.1	Incorrect
	Yes	681	73.9	Correct

Table S4: Factors significantly affecting knowledge scores among enrolled control group participants (N=461) & CVD patients (N=460) in this study

CVD Knowledge score		**Non-CVD (CG)**				**CVD**			
		N	Mean	Std. Deviation	P.value	N	Mean	Std. Deviation	P.value
**Gender**	Male	220	23.55	7.99	0.930	242	26.81	5.35	0.334
	Female	241	23.49	7.84		218	26.34	4.95	

**Age**	< 45	318	23.84	8.11	0.158	60	26.17	5.89	**0.037**
	45 - 65	118	23.25	6.90		253	27.14	4.73	
	> 65	25	20.76	9.33		147	25.82	5.47	
**Marital Status**	Single	219	24.11	8.28	**0.003**	39	27.21	5.16	**0.033**
	Married	220	23.30	7.24		357	26.75	5.17	
	Divorced	10	24.80	8.39		13	27.77	4.49	
	Widowed	12	15.67	8.80		51	24.67	4.97	
**Working Status**	Not working	136	21.75	8.61	**0.000**	86	26.34	5.57	**0.000**
	Healthcare professional	70	30.51	5.28		24	31.17	4.04	
	Non-healthcare professional	192	22.25	7.20		197	26.32	5.15	
	Household	63	23.44	6.65		153	26.35	4.80	
**Governorates**	Beirut	73	25.95	6.21	0.068	80	27.54	4.85	**0.017**
	North Lebanon	89	22.63	8.76		43	26.35	4.98	
	South Lebanon	92	23.36	7.62		75	24.87	6.60	
	Mount Lebanon	105	22.92	8.68		219	26.77	4.61	
	Beqaa	102	23.32	7.41		43	27.14	5.24	
**Educational level**	No formal education	12	16.33	9.87	**0.002**	24	22.75	6.35	**0.000**
	Elementary class	11	24.82	4.92		93	25.09	5.19	
	Complementary	35	23.09	7.05		91	26.40	4.37	
	Secondary	71	21.35	6.67		98	27.17	4.95	
	University level	266	24.26	8.11		118	27.43	4.89	
	Postgraduate	66	24.20	7.87		36	29.17	5.40	
**Monthly Income**	0-750,000LL	157	22.54	8.15	**0.014**	124	26.30	5.35	0.139
	751,000-1,500,000LL	116	23.32	7.50		91	25.80	5.79	
	1,501,000-3,000,000LL	97	23.49	7.67		91	26.73	4.59	
	3,001,000-4,500,000LL	39	23.51	7.72		59	27.98	4.82	
	More than 4,500,000LL	52	26.98	7.95		95	26.73	4.91	
**Smoking**	No	326	24.20	7.85	**0.004**	257	27.47	4.97	**0.000**
	Yes	135	21.88	7.82		203	25.48	5.20	

Table S5: Factors significantly affecting attitude scores among enrolled control group participants (N=461) & CVD patients (N=460) in this study

CVD Attitude score		Non-CVD (CG)				CVD			
		N	Mean	Std. Deviation	P-Value	N	Mean	Std. Deviation	P-Value
**Gender**	Male	220	59.99	12.67	**0.022**	242	63.06	9.81	0.569
	Female	241	62.63	12.08		218	63.60	10.63	
**Age**	< 45	318	61.86	12.62	0.266	60	65.22	11.14	**0.016**
	45 - 65	118	60.77	12.17		253	63.97	9.73	
	> 65	25	57.96	10.75		147	61.41	10.37	
**Marital Status**	Single	219	61.99	13.03	0.264	39	64.74	10.69	0.638
	Married	220	61.06	11.92		357	63.35	10.08	
	Divorced	10	62.40	9.98		13	63.54	9.66	
	Widowed	12	54.92	11.20		51	61.94	10.91	
**Working Status**	Not working	136	61.74	12.07	**0.035**	86	61.97	10.07	0.427
	Healthcare professional	70	64.84	13.29		24	64.63	9.33	
	Non-healthcare professional	192	59.82	12.28		197	63.95	9.73	
	Household	63	61.43	12.06		153	63.05	10.97	
**Governorates**	Beirut	73	61.56	12.59	0.626	80	65.30	10.06	**0.024**
	North Lebanon	89	60.80	13.06		43	64.91	10.94	
	South Lebanon	92	63.14	12.23		75	63.07	11.28	
	Mount Lebanon	105	60.92	12.03		219	61.87	9.70	
	Beqaa	102	60.59	12.38		43	65.84	9.32	
**Educational level**	No formal education	12	53.17	9.06	**0.004**	24	57.38	11.71	**0.002**
	Elementary class	11	53.09	11.92		93	61.12	10.02	
	Complementary	35	57.46	9.10		91	62.71	10.30	
	Secondary	71	60.79	13.36		98	64.73	9.39	
	University level	266	62.15	12.70		118	65.10	9.76	
	Postgraduate	66	63.77	11.06		36	64.75	10.85	
**Monthly Income**	0-750,000LL	157	61.40	12.28	**0.003**	124	62.57	11.30	0.497
	751,000-1,500,000LL	116	58.35	13.29		91	62.79	10.33	
	1,501,000-3,000,000LL	97	62.10	12.59		91	63.75	8.11	
	3,001,000-4,500,000LL	39	61.62	9.69		59	62.61	12.14	
	More than 4,500,000LL	52	66.44	10.78		95	64.81	9.00	
**Smoking**	No	326	62.83	12.16	**0.000**	257	64.98	10.10	**0.000**
	Yes	135	57.84	12.39		203	61.20	9.95	

Table S6: Factors significantly affecting practice scores among enrolled control group participants (N=461) & CVD patients (N=460) in this study

CVD Practice score		N	Mean	Std. Deviation	P.value	N	Mean	Std. Deviation	P.value
**Gender**	Male	220	5.80	2.25	**0.000**	242	6.04	1.77	0.607
	Female	241	6.74	2.11		218	5.95	1.68	
**Age**	< 45	318	6.42	2.21	0.173	60	6.30	1.64	0.157
	45 - 65	118	5.98	2.24		253	6.04	1.73	
	> 65	25	6.12	2.22		147	5.81	1.75	
**Marital Status**	Single	219	6.53	2.13	0.154	39	6.44	1.71	0.058
	Married	220	6.11	2.29		357	6.03	1.75	
	Divorced	10	5.90	2.18		13	5.69	1.32	
	Widowed	12	5.67	2.46		51	5.49	1.58	
**Working Status**	Not working	136	6.20	2.06	**0.011**	86	5.86	1.81	0.760
	Healthcare professional	70	7.06	2.00		24	6.04	1.99	
	Non-healthcare professional	192	6.05	2.30		197	6.09	1.71	
	Household	63	6.40	2.39		153	5.95	1.67	
**Governorates**	Beirut	73	6.60	2.24	**0.043**	80	6.20	1.71	**0.012**
	North Lebanon	89	6.52	1.87		43	5.81	1.45	
	South Lebanon	92	6.61	1.96		75	5.88	1.69	
	Mount Lebanon	105	5.83	2.43		219	5.84	1.75	
	Beqaa	102	6.07	2.43		43	6.79	1.75	
**Educational level**	No formal education	12	5.33	2.61	**0.008**	24	5.54	1.82	**0.012**
	Elementary class	11	4.73	2.69		93	5.47	1.66	
	Complementary	35	5.77	2.22		91	6.19	1.78	
	Secondary	71	6.01	2.24		98	6.12	1.77	
	University level	266	6.41	2.11		118	6.14	1.58	
	Postgraduate	66	6.83	2.31		36	6.39	1.81	
**Monthly Income**	0-750,000LL	157	6.11	2.12	0.368	124	5.98	1.74	0.810
	751,000-1,500,000LL	116	6.27	2.39		91	5.91	1.76	
	1,501,000-3,000,000LL	97	6.41	2.35		91	6.20	1.71	
	3,001,000-4,500,000LL	39	6.90	1.93		59	5.97	1.88	
	More than 4,500,000LL	52	6.23	2.09		95	5.94	1.62	
**Smoking**	No	326	6.66	2.08	**0.000**	257	6.43	1.64	**0.000**
	Yes	135	5.41	2.33		203	5.45	1.68	

Table S7: Multiple linear regression results showing predictors of KAP about CVD among enrolled control group participants (N=461) & CVD patients (N=460) in this study

Variables	Group of participants	Predictors	Standardized Coefficient (β)	P-value
Knowledge score	**Non-CVD (CG)**	(Constant)	22.573	0.000
		Working as healthcare professional	7.751	0.000
		Widowed	-7.438	0.000
		Beirut	2.610	0.005
		Smoking: Are you a current smoker?	-1.533	0.039
	**CVD**	(Constant)	24.962	0.000
		Degree of educational attainment	0.837	0.000
		Smoking: Are you a current smoker?	-1.808	0.000
		Working as healthcare professional	3.743	0.000
		South Lebanon	-1.419	0.023
		Beirut	1.378	0.024
Attitude score	**Non-CVD (CG)**	(Constant)	51.022	0.000
		Degree of educational attainment	1.675	0.002
		Knowledge	0.226	0.002
		Smoking: Are you a current smoker?	-3.729	0.003
	**CVD**	(Constant)	49.794	0.000
		Knowledge	0.511	0.000
		Mount Lebanon	-3.211	0.000
		Degree of educational attainment	0.969	0.004
		Smoking: Are you a current smoker?	-2.543	0.005
Practice score	**Non-CVD (CG)**	(Constant)	2.620	0.000
		Attitudes	0.048	0.000
		Smoking: Are you a current smoker?	-0.843	0.000
		Gender	0.674	0.001
		Mount Lebanon	-0.715	0.002
		Working as healthcare professional	0.637	0.016
	**CVD**	(Constant)	2.151	0.000
		Attitudes	0.066	0.000
		Smoking: Are you a current smoker?	-0.730	0.000
		Beqaa	0.685	0.005
		Widowed	-0.527	0.018
